# Author Correction: α-Synuclein emulsifies TDP-43 prion-like domain—RNA liquid droplets to promote heterotypic amyloid fibrils

**DOI:** 10.1038/s42003-023-05762-6

**Published:** 2024-01-09

**Authors:** Shailendra Dhakal, Malay Mondal, Azin Mirzazadeh, Siddhartha Banerjee, Ayanjeet Ghosh, Vijayaraghavan Rangachari

**Affiliations:** 1 Department of Chemistry and Biochemistry, School of Mathematics and Natural Sciences, University of Southern Mississippi, Hattiesburg, MS 39406 USA; 2 Center for Molecular and Cellular Biosciences, University of Southern Mississippi, Hattiesburg, MS 39406 USA; 3 Department of Chemistry and Biochemistry, University of Alabama, Tuscaloosa, AL 35401 USA

**Keywords:** Supramolecular assembly, Electron microscopy

Correction to: *Communications Biology* 10.1038/s42003-023-05608-1, published online 05 December 2023.

In the original version of the Article, references to colors found in some charts were omitted from the Results and from the legend for Figure 3c.

The first paragraph of the Results should read:

“Fluorescence resonance recovery after photobleaching (FRAP) analysis on the colocalized puncta presumed to be SGs, showed partial recovery (~60%) with TDP-43PrLD, suggesting diminished internal mobility or in other words, gelation (red, ▪; Fig. 1b). In contrast, αS showed no recovery in these puncta which suggests that it could be in an aggregated state (green, ●; Fig. 1b). Under non-stressed conditions, FRAP on the colocalized puncta showed significant attenuation of recovery for TDP-43PrLD (~ 40%) (red, ▪; Fig. 1d) while αS did not show any (green, ●; Fig. 1d).”

The second paragraph of the Results should read:

“As expected, the turbidity values increase with the increase in RNA concentrations and peak at 75 µg/mL in the absence of αS (grey, ●; Fig. 3c). Changes in αS concentrations did not alter this pattern to any significant extent but marginally increased the turbidity values between 50 and 100 µg/mL RNA (blue, green & red, Fig. 3c).”

And further down in the same paragraph of the Results should read:

“Equimolar incubations with 10 μM αS and TDP-43PrLD showed only 19% of total αS partitioning in the droplets (red; Fig. 3g), while sub-stoichiometric (5 μM) and excess (20 μM) αS showed 17% (blue and green; Fig. 3g) and were statistically insignificant from one another. The partitioning of αS with insignificant quantitative change despite stoichiometric variance could be attributed to the protein’s preferential localization on the surface of preformed TDP-43PrLD droplets preventing further recruitment from the bulk/dilute phase. Next, the degree of partitioning was determined as a function of RNA concentrations which showed a negative correlation between the two; 50 μg/mL of RNA (red) showed maximal partitioning with 19% while incubations with 100 and 150 μg/mL of RNA showed 12 and 8%, respectively (green and blue; Fig. 3h), suggesting charge-repulsion as a possible contributor for partitioning. To see what sequence determinants in αS are responsible for partitioning into TDP-43PrLD – RNA droplets, truncation constructs αS∆NTD and αS∆NAC were used. The N-terminal domain (NTD; 1-60) is highly charged and the central amyloid region (NAC; 61-95) is rich in hydrophobic residues therefore, deletion of these regions could provide clues about the contributions of electrostatic and hydrophobic forces, respectively. Both truncation mutants decreased the amount of αS partitioning by half (~9%) compared to the wild-type (red; Fig. 3i). Kinetics of partitioning, however, showed differences with αS ∆NTD displaying first-order exponential rate and αS ∆NAC showing second-order fits containing rapid and slower kinetic events with an overall slower rate than αS ∆NTD (cyan and orange; Fig. 3i).”

The fifth paragraph of the Results should read:

“The sample containing αS showed an increase in ThT from 20 hours (red; Fig. 6c) as opposed to the sample without αS which did not show an increase in ThT intensity even after 50 hours (black; Fig. 6c). The observed lag time could be due to the combined effects of αS-seeded TDP-43PrLD aggregation as well as the time taken to ThT to permeate into the droplets. Incubation of the sedimented droplets in the presence of 5 μM TDP-43PrLD monomers further exacerbated the lag time difference between the droplets with and without αS. The samples with αS showed a lag time of 8-9 hours (red; Fig. 6d) while the ones in its absence showed a lag time of ~25 hours (black; Fig. 6d).”

The legend for Figure 3c should read:

“Turbidity measurements based on absorbance measured at 600 nm on 10 µM TDP-43PrLD samples with increasing poly-A RNA concentrations (0-200 µg/mL) and increasing αS concentrations (grey, 0 µM; green, 5 µM; red, 10 µM, and blue, 20 µM), (n = 3).”

This has now been corrected in the PDF and HTML versions of the Article.

