# Supplementary material for: α-Synuclein emulsifies TDP-43 prion-like domain—RNA liquid droplets to promote heterotypic amyloid fibrils
Source: Commun Biol. 2023 Dec 5;6:1227. doi: 10.1038/s42003-023-05608-1 (PMC10697960; doi:10.1038/s42003-023-05608-1)
Supplement: Supplementary file 2 — Supplementary Information [file 42003_2023_5608_MOESM2_ESM.pdf]

α-Synuclein emulsifies TDP-43 prion-like domain – RNA liquid droplets to promote heterotypic amyloid fibrils

Shailendra Dhakal[1,2], Malay Mondal[1,2], Azin Mirzazadeh[1,2], Siddhartha Banerjee[3], Ayanjeet Ghosh[3], and Vijayaraghavan Rangachari[1,2]*

SUPPLEMENTARY INFORMATION

SUPPLEMENTARY FIGURES

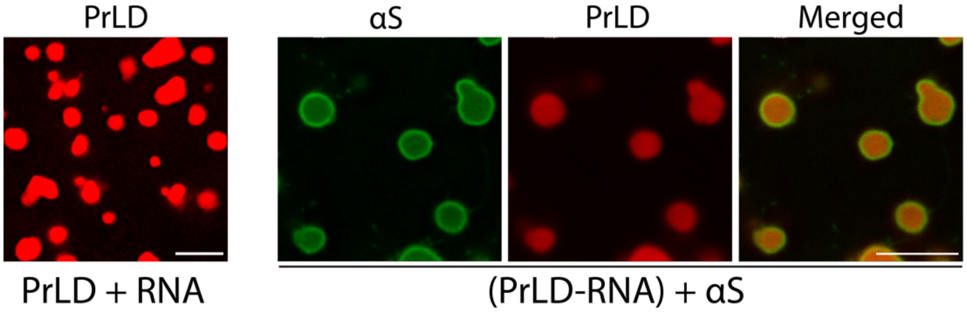

Figure S1. Confocal images showing the condensates of histidine tag-cleaved TDP-43PrLD and RNA with and without histidine tag-cleaved αS monomers. Scale bar = 5 microns.

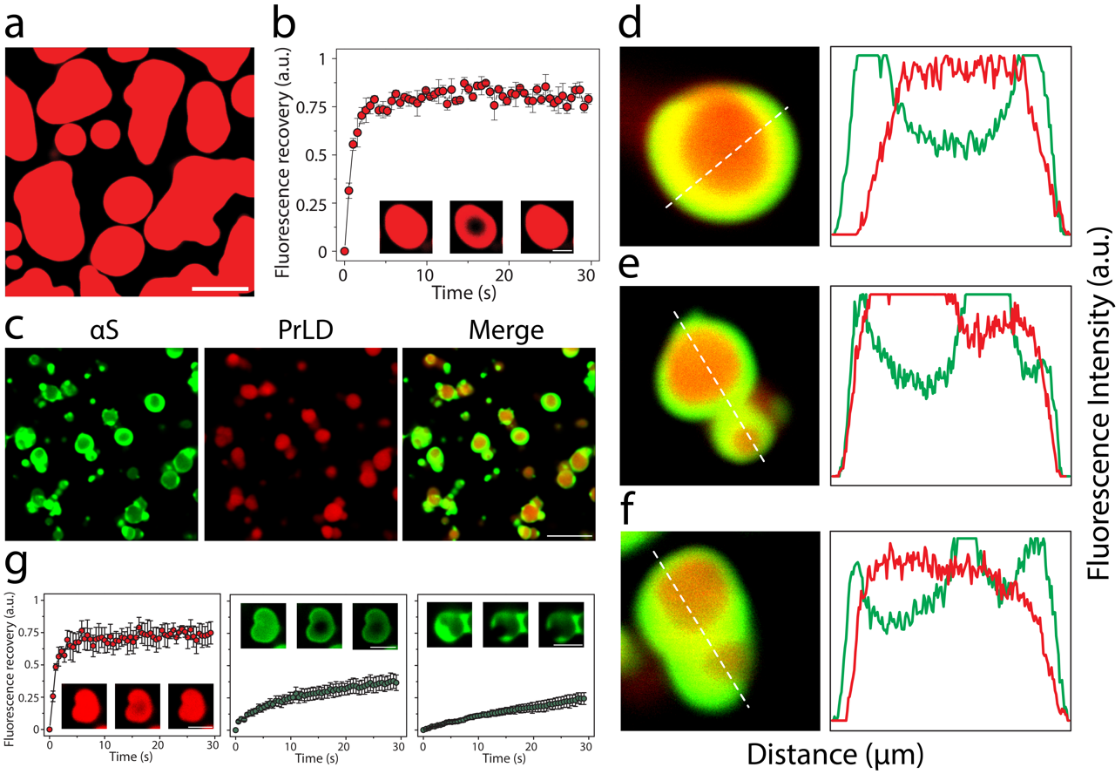

Figure S2. αS monomers influence on TDP-43PrLD – RNA condensates at 6 hours. **a-b** Images showing TDP-43PrLD – poly-A RNA condensates at 6 hours of incubation **(a)**, and its FRAP kinetics plotted as a function of time **(b)**. Images in the inset indicate pre-bleach, during bleach, and post-bleach states, respectively. **c** Images showing partitioning of αS in the pre-formed TDP-43PrLD – RNA liquid droplets with TDP-43PrLD (red), αS (green), and overlay (yellow). **d-f** Partitioning analysis of αS based on fluorescence intensity of the droplets from (**c**). Intensity plot against the distance corresponds to the white dotted line drawn through the droplets. **g** FRAP data on TDP-43PrLD (left), αS at the center (middle), and αS at the periphery (right) from the samples in (**c**) (n = 3). Images in the inset indicate pre-bleach, during bleach, and post-bleach states, respectively.

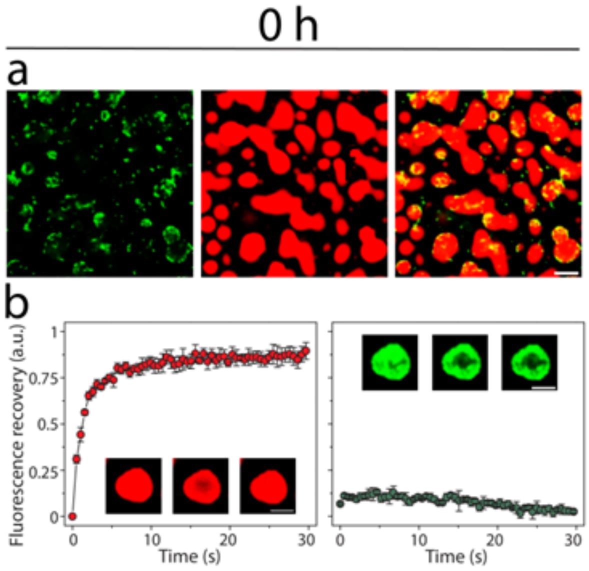

Fig S3. Effect of αS fibrils on the TDP-43PrLD – RNA condensates. **a-b** Confocal microscopy images of TDP-43PrLD – RNA preformed droplets upon the addition of αS fibrils at 0 hours (**a**), FRAP data showing the recovery of the TDP-43PrLD (left) and αS fibrils (right) **(b)** Insets indicate pre-bleach, bleach, and post-bleach images (left to right). **c-d** The same reaction at (a) is imaged and subjected to FRAP at 24 hours. Scale bar of images = 5 µm, FRAP insets = 2 µm.

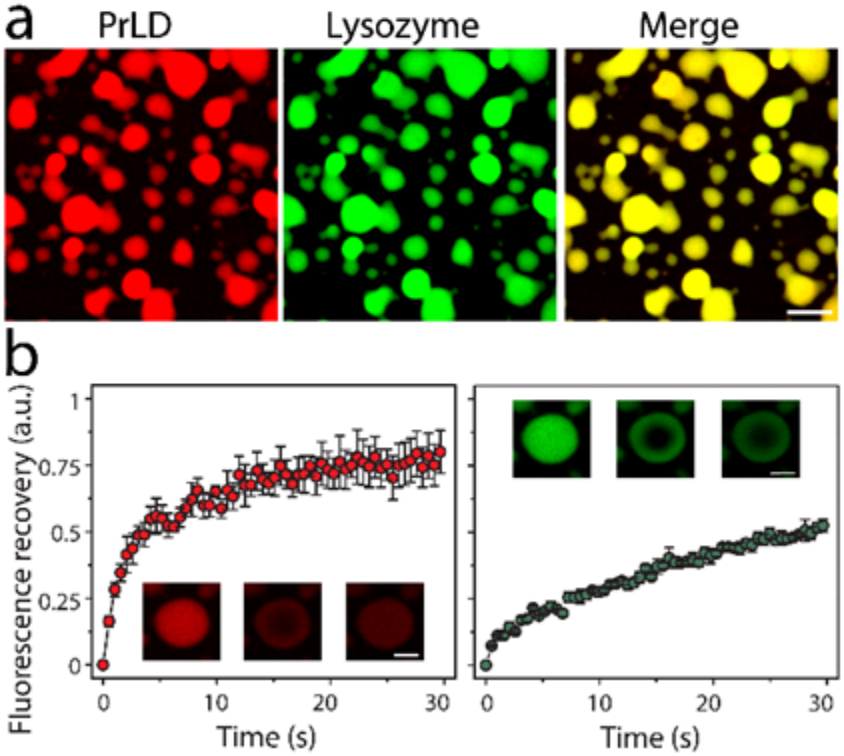

Fig S4. Lysozyme partitioning on the TDP-43PrLD and RNA droplets. **a** Images showing the partitioning of lysozyme on the preformed TDP-43PrLD and RNA condensates **b** FRAP recovery of TDP-43PrLD (left) and lysozyme (right) from the reaction on (a). Insets represent pre-bleached, bleached and post-bleached droplets (left to right).

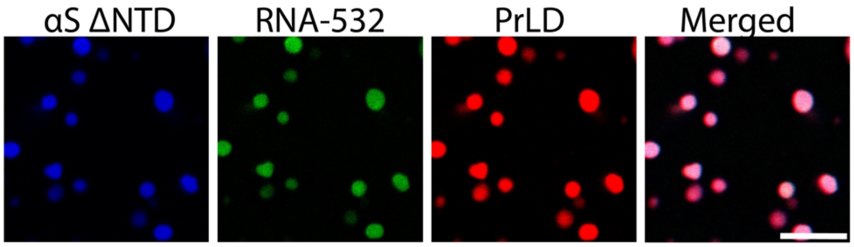

Figure S5. Representative confocal images of fluorescently labeled TDP-43PrLD and RNA droplets upon the addition of alpha-synuclein ΔNTD. Scale bar = 5 μM.

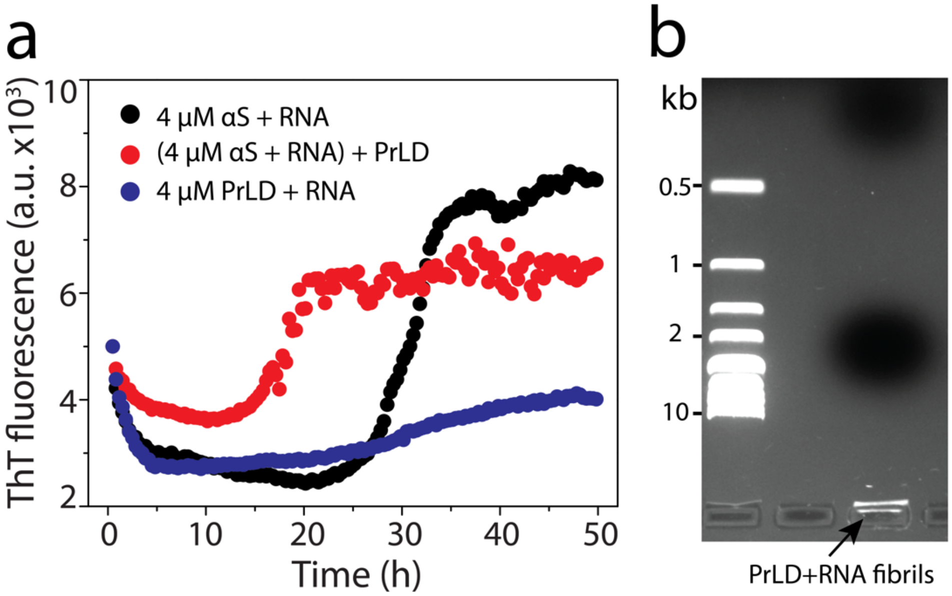

Fig S6. Influence of RNA in aggregation. a) ThT fluorescence showing the effect TDP-43PrLD on the aggregation of αS – RNA mixture along with αS / TDP-43PrLD – RNA as a control. b) Ethidium bromide staining of the TDP-43PrLD fibrils isolated from the condensates of TDP-43PrLD and RNA at 72 hours of reaction.

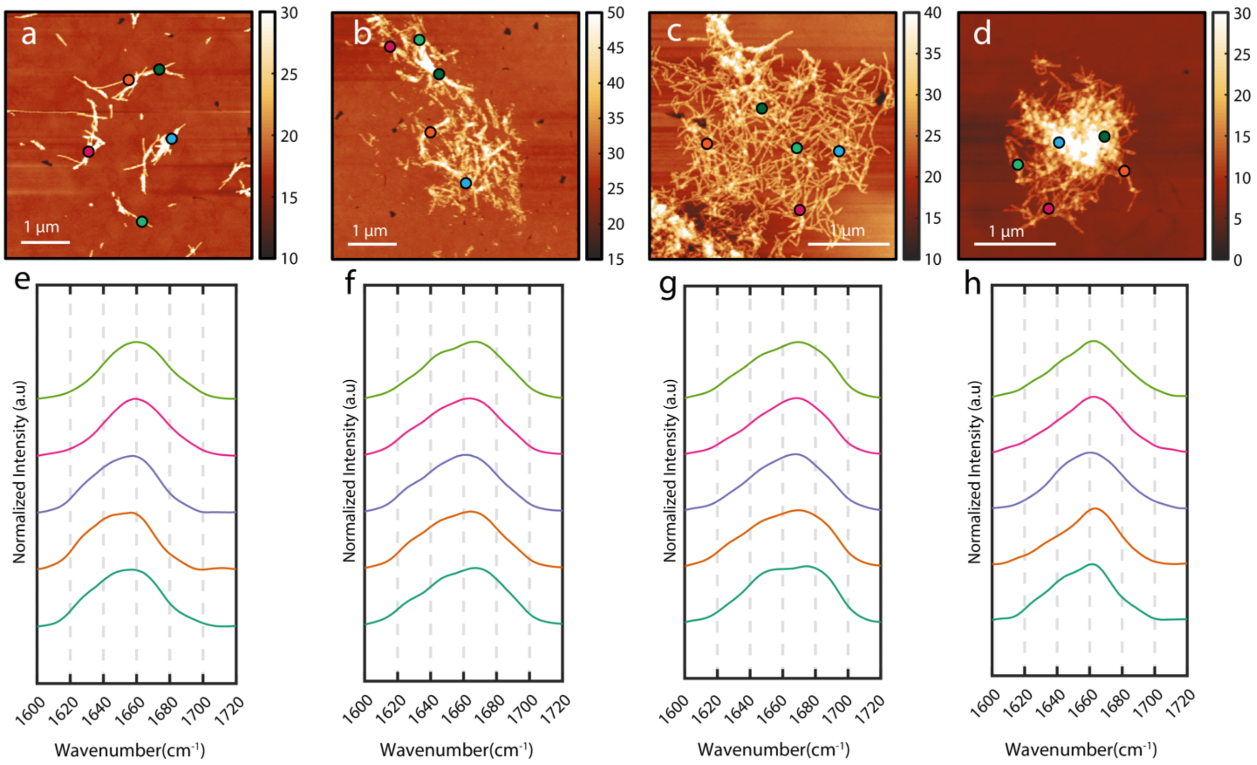

Fig S7: AFM-IR analysis. Representative spatial locations (shown in circles) on fibrils from where the IR spectra are recorded for (a) alpha-synuclein, (b) TDP -43PrLD, (c) TDP -43PrLD with no alpha-synuclein in droplet and (d)TDP -43PrLD with alpha-synuclein containing droplet. The bottom row shows representative spectra from each sample which are color coded with their spatial locations.
